# Evaluating Lower Computational Burden Approaches for Calibration of Large Environmental Models

**DOI:** 10.1111/gwat.13106

**Published:** 2021-06-08

**Authors:** Randall J. Hunt, Jeremy T. White, Leslie L. Duncan, Connor J. Haugh, John Doherty

**Affiliations:** ^1^ Intera Inc. Fort Collins CO USA; ^2^ U.S. Geological Survey Lower Mississippi Gulf Water Science Center Nashville TN USA; ^3^ Watermark Numerical Computing Brisbane Queensland Australia

## Abstract

Realistic environmental models used for decision making typically require a highly parameterized approach. Calibration of such models is computationally intensive because widely used parameter estimation approaches require individual forward runs for each parameter adjusted. These runs construct a parameter‐to‐observation sensitivity, or Jacobian, matrix used to develop candidate parameter upgrades. Parameter estimation algorithms are also commonly adversely affected by numerical noise in the calculated sensitivities within the Jacobian matrix, which can result in unnecessary parameter estimation iterations and less model‐to‐measurement fit. Ideally, approaches to reduce the computational burden of parameter estimation will also increase the signal‐to‐noise ratio related to observations influential to the parameter estimation even as the number of forward runs decrease. In this work a simultaneous increments, an iterative ensemble smoother (IES), and a randomized Jacobian approach were compared to a traditional approach that uses a full Jacobian matrix. All approaches were applied to the same model developed for decision making in the Mississippi Alluvial Plain, USA. Both the IES and randomized Jacobian approach achieved a desirable fit and similar parameter fields in many fewer forward runs than the traditional approach; in both cases the fit was obtained in fewer runs than the number of adjustable parameters. The simultaneous increments approach did not perform as well as the other methods due to inability to overcome suboptimal dropping of parameter sensitivities. This work indicates that use of highly efficient algorithms can greatly speed parameter estimation, which in turn increases calibration vetting and utility of realistic models used for decision making.

## Introduction

The need for fast‐running representative simulations has become critical as models move to decision making contexts such as integrated water management (e.g., Jakeman et al. 2016) and data assimilation. Representative environmental simulations typically involve a highly parameterized approach (e.g., Moore and Doherty 2005; Hunt et al. 2007; Doherty and Hunt 2010; Knowling et al. 2019), and often incorporate transience—both of which can add appreciably to forward model and associated parameter estimation (PE) runtimes.

Approaches to speed PE are important because standard approaches require many forward model runs—sometimes tens of thousands of runs or more—to determine a quantifiable best fit to an inverse problem. The higher computational burden results from derivative‐based algorithms requiring a parameter sensitivity matrix (i.e., the Jacobian matrix) to determine an efficient set of potential parameter upgrades. The size of the Jacobian matrix is the number of observations (rows) times the number of parameters (columns). Because it is a sensitivity matrix, it is most commonly filled by calculating the difference between a base run calculated at initial parameter values and results from a forward model run with a single perturbed parameter value using a finite‐difference design to approximate the sensitivity near the initial parameter value. In this approach, an initial forward run and a series of perturbed forward runs are performed where each individual calibration parameter is changed by a small amount (usually 1%) and the model is run while all other parameters are held constant at their initial values. In this way, any changes in the observation values can be directly attributed to changes in the perturbed parameter.

Perturbation is typically performed using a “forward‐difference” approach (parameters perturbed by addition of a small increment) in early PE iterations then switching to a higher precision “central‐difference” approach (parameters are perturbed by addition and subtraction of a small increment) in later iterations when expected improvement is more modest because the model to measurement misfit is nearly optimal. The number of forward runs needed to fill the matrix equals the number of adjustable parameters plus one (i.e., the initial unperturbed run) for the forward‐difference case and almost double that for the central‐difference case. This computational burden is compounded because the Jacobian matrix is typically recalculated in an iterative scheme each time a new updated parameter set is selected from the set of potential parameter upgrades, which then becomes the base parameter set for the next PE iteration. That is:

“A first update to initial parameter values does not complete the parameter estimation process because the groundwater inverse problem is nonlinear and sensitivities contained in the initial Jacobian matrix cannot accurately represent the sensitivities of the solution using the new parameter values. Therefore, a new Jacobian matrix is calculated using the parameters that gave the lowest objective function, which becomes the new unperturbed base case…” (Anderson et al. 2015, 406).

Therefore, following this strategy, the computational expense of calculating the Jacobian matrix must be incurred each PE iteration when a new potential parameter upgrade is calculated.

There are less computationally demanding alternatives to the finite‐difference approach for filling the Jacobian matrix. Solving the PE problem in terms of superparameters (linear combinations of base parameters—Tonkin and Doherty 2005) can reduce the PE computational burden. This approach requires an initial full base‐parameter Jacobian (sensitivity for every adjustable parameter) to define a smaller set of superparameters, after which only superparameter sensitivities are needed to calculate potential parameter upgrades. However, depending on the results of the PE analysis, a full Jacobian may need to be rerun, superparameters redefined, and the PE effort restarted, to ensure that nonlinearity in the PE problem is accounted for (e.g., Welter et al. 2012, 2015; White et al. 2020).

Other less computationally intensive methods include “direct sensitivity” calculation such as used in MODFLOW‐2000 (Hill et al. 2000) where equations that result from differentiating the discretized governing equations are solved internally within the model code and those obtained using specialized forward model codes that use an adjoint state formulation of the forward model (e.g., Sykes et al. 1985). These methods represent a more accurate sensitivity calculation, but are relatively inflexible in that they generally require special versions of the forward model code, and can only calculate sensitivities for parameter and observation types within the code itself. That is, support for derived and processed observations (which can be important for defining a prediction‐specific objective function or accommodating model structural imperfections) generally must be coded within the forward model solution algorithm. These issues can make implementation and upkeep of such modeling codes problematic for integrated water management. Moreover, such limitations in applicability obviate the universality of a PE approach.

**Figure 1:**
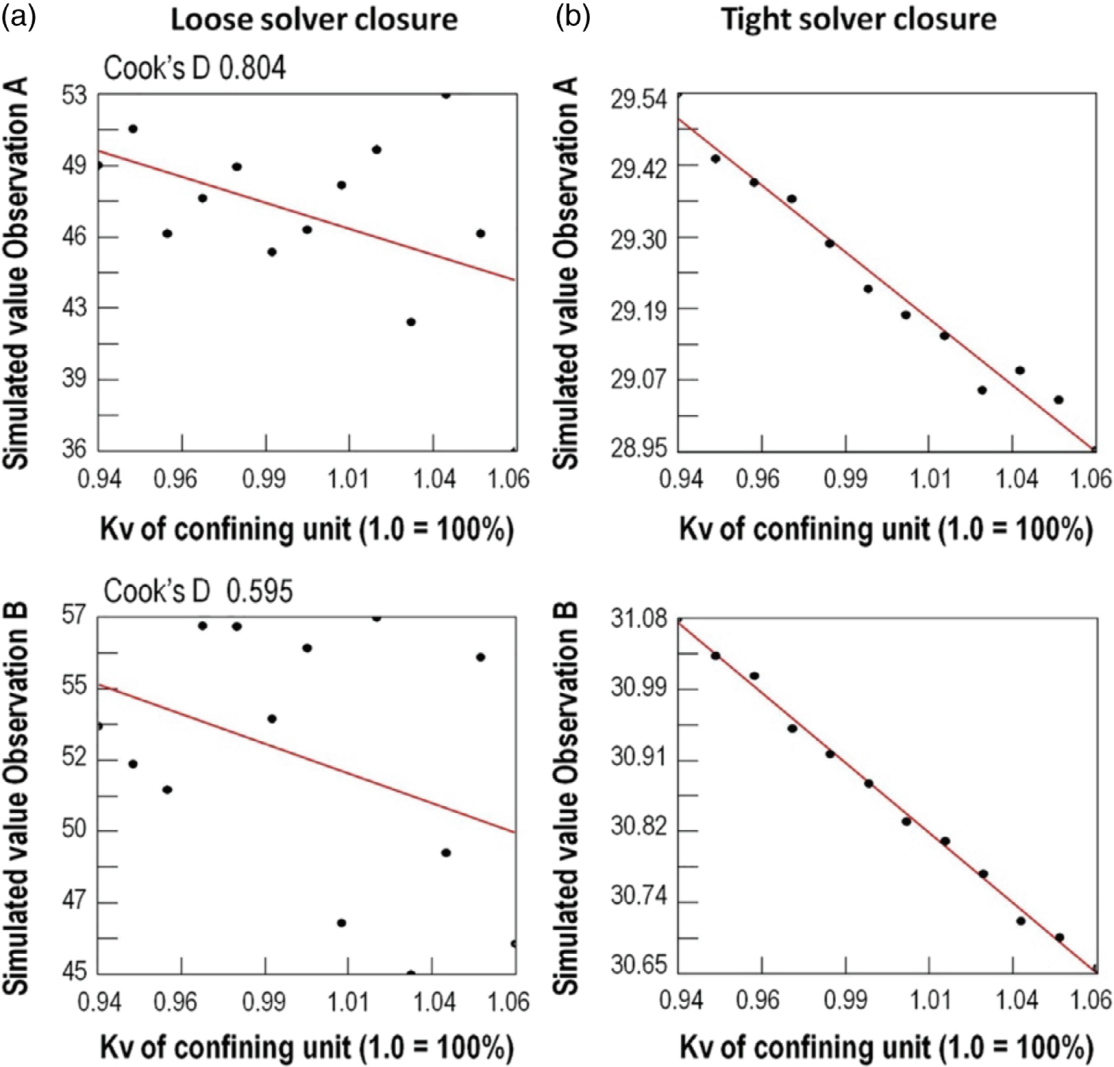
Plot of change in model outputs (*y*‐axes) to small increments of change in one model parameter (vertical hydraulic conductivity, Kv, on the *x*‐axes) for two different observations. Each dot represents one model run; the straight line is the best fit through the dots. Because the true parameter sensitivity derivative is approximated using a 1% parameter perturbation, sequential 1% perturbations are expected to provide a coherent change (e.g., the monotonically changing line shown in (b)). Poor derivatives calculated by perturbation (a) can confound derivative‐based parameter estimation methods; tighter solver closure as shown in (b) provides more coherent derivatives. An influence statistic (Cook's *D*) for the two observations is also listed, where higher values represent more influence on the regression (modified from Feinstein et al. 2008; Anderson et al. 2015).

Even though a perturbation‐based sensitivity only approximates the actual local derivative of an observation with respect to a parameter at one point in parameter space, it has been found to be sufficiently accurate for applied modeling (Yager 2004). The widespread suitability of approximate parameter sensitivity approaches might seem surprising when the actual quality of the derivative is evaluated. For example, the PEST software suite includes the JACTEST utility, which evaluates simulated values (needed for sensitivity calculation) at various multiples of perturbation (Figure 1). Derivative‐based PE methods work best when there is a monotonic, or at least coherent, relation between successive perturbation increments and a simulated value (Figure 1b), yet many times the derivatives are incoherent (Figure 1a), where successive perturbations can result in increasing or decreasing observed values. In almost all cases the incoherence shown in Figure 1 is not describing the real world, or the mathematical characterization of the real world, in a model. Rather they reflect numerical artifacts that arise within the computer code—artifacts that are rarely evaluated in practice.

PE success with perturbation methods despite such potential noise in the sensitivities may be due to the difference of observation importance for the PE problem. This relation has been described in terms of statistical influence (Yager 1998; Hunt et al. 2006; Hill and Tiedeman 2007). Adequate derivatives relating to observations with high statistical influence (e.g., observations with high statistical leverage or high Cook's *D* statistic) can have enough signal to overcome the noise in sensitivities of less influential observations. Such widespread applicability of approximate sensitivities indicates a larger robustness and tolerance to approximate sensitivities, which can be exploited to reduce the computational burden of PE efforts. That is, sensitivities for PE applications can be thought of as simply a “means to an end,” where the sensitivity “means” provide the basis for calculating an informed set of potential parameter upgrades, but the “end”—a parameter set that provides a better fit—is what is ultimately important. High degrees of parameter sensitivity approximation (with associated reduced computational burden), therefore, may still provide sufficient information to efficiently identify candidate parameter upgrades for testing.

Here methods for reducing the PE computational burden were evaluated using a groundwater model used for decision making in the Mississippi Alluvial Plain, USA. For comparison, a standard and computationally expensive approach was compared to three other PE efforts that used more approximate and less computationally expensive approaches. The less computationally demanding approaches involved (1) simultaneous increments; (2) an iterative ensemble smoother; and (3) randomized Jacobians. The first and third approaches are available within PEST‐HP (Doherty 2020); the second is part of the PEST++ Version 5 (White et al. 2020) software suite.

## Methods

The four PE approaches were applied to the same groundwater forward model with the same adjustable parameters, parameter bounds, PE closure criteria, observations, observation weights, template files and instruction files. Therefore, our test focused on the application of the PE algorithms. For each PE approach, additional and optional control variables used reasonable default values arising from experience of the authors. These default values were not tuned after initial selection and are listed in Appendix S1. The groundwater model and calibration setup are briefly described here and also in more detail in Appendix S1. Theoretical descriptions for the PE approaches are described in detail elsewhere but are also briefly discussed here; the presentation here emphasizes the application and performance of each PE approach. Our investigation focused on number of forward runs, acceptable fit to observations, plausibility of estimated parameters, similarity to existing approaches users are already familiar with, and ability to leverage uncertainty analysis approaches.

### Groundwater Forward Model and Calibration Setup

**Figure 2:**
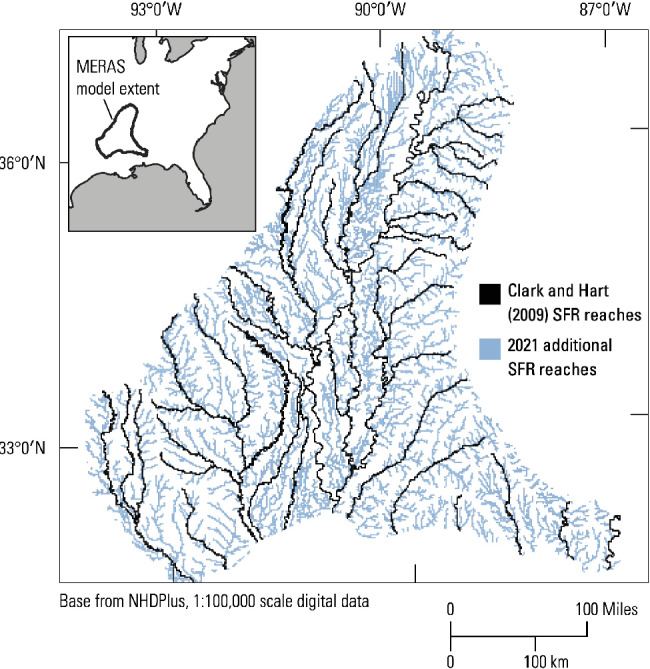
Extents and surface‐water system in Clark and Hart (2009) and the updated model used in this study. MERAS, Mississippi Embayment Regional Aquifer Study; SFR, stream flow routing.

A complex model representing a societally relevant hydrologic system was chosen for testing in order to better illustrate the applicability of the candidate PE approaches to real world problems. The groundwater model leveraged previously published 13‐layer models from the Mississippi Embayment regional aquifer system, USA, which are discussed in more detail in the Appendix S1. The underlying model extents (Figure 2) and spatial discretization are described by Clark and Hart (2009). A series of updates to the existing modeling were performed to provide more current forecasts for stakeholders, including (1) higher stream density; (2) more spatially refined recharge array; (3) better estimates of water use; (4) more recent time period simulated; (5) more realistic storage conceptualization; and (6) more robust handling of dry nodes. Appendix S1 describes the development of these updates more fully; all model files are also available online (Hunt et al. 2021).

The resulting updated model uses 16 stress periods and the computer code MODFLOW‐NWT (Niswonger et al. 2011). Initial dynamic equilibrium is simulated using pumping representing long‐term rates (of which 1998 to 2007 average rates are representative). This is followed by 7 stress periods of spin‐up and 8 stress periods for calibration. Following the temporal discretization of Clark and Hart (2009), a stress period covers a 6‐month growing or nongrowing season period. The spin‐up period extends from 1 October 2008 to 31 March 2012; the calibration period extends from 1 April 2012 to 31 March 2016. The resulting forward model run had a >6 h runtime on a modern desktop.

**Figure 3:**
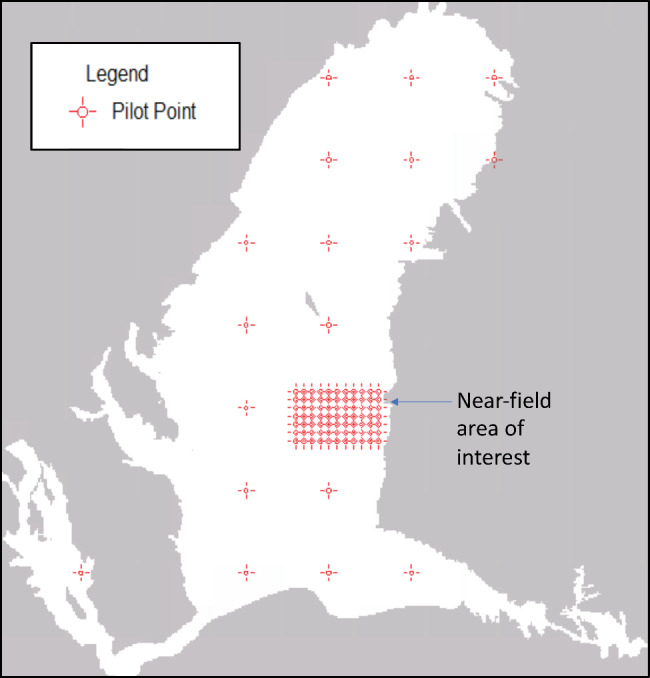
Layer 2 extents and location of hydraulic conductivity and specific yield pilot points.

**Figure 4:**
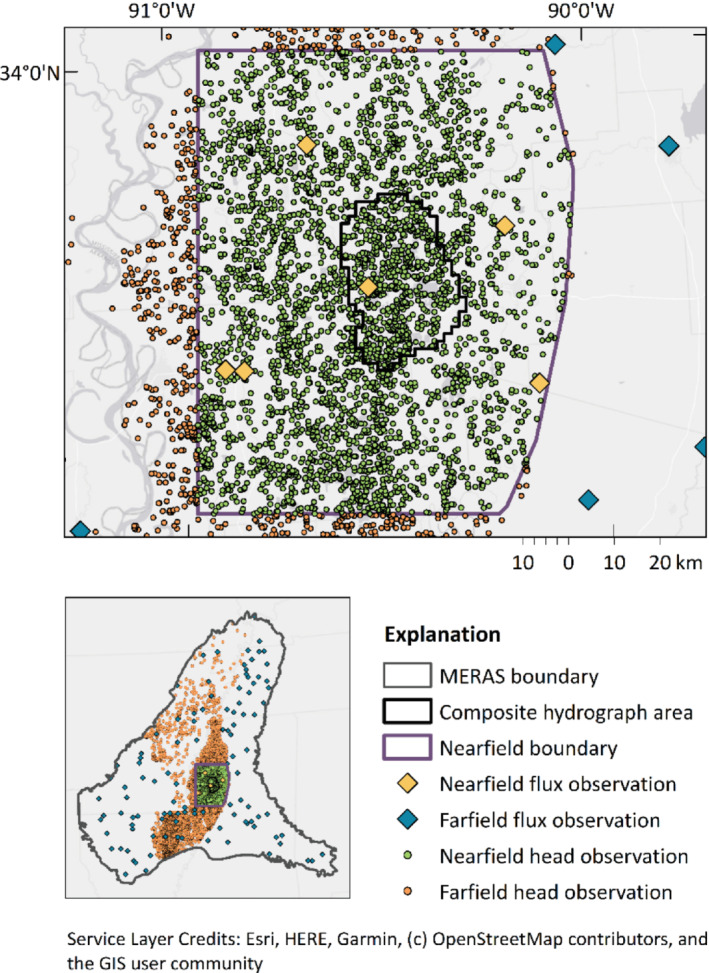
Farfield and nearfield (inset) calibration target locations. MERAS, Mississippi Embayment Regional Aquifer Study.

The observations included hydraulic head, hydraulic head difference, flux, and flux difference targets for the spin‐up and calibration periods. To better reflect the scale that future models in the system would be applied, the PE effort focused on a subset rather than the entirety of the Clark and Hart (2009) multi‐state model domain. Thus, the PE domain consisted of a “nearfield” that included an area of interest to an identified stakeholder group, and a “farfield” consisting of the rest of the model domain (Figures 3 and 4). Only calibration‐period observations in the nearfield were included as weighted targets for PE (Figure 4). This is one approach for weighting (e.g., farfield targets could be given lesser‐but‐not‐zero weight), and underscores our focus of testing PE algorithms on scales more typical for applied hydrogeological questions. How weights were assigned is described in more detail in Appendix S1. For PE testing purposes, the salient point is that the same weights were assigned to the same calibration targets for each algorithm tested. A total of 248,398 observations were included whereby 30,288 were weighted for calibration. A high number of observations is expected to be more common as the ability to measure the natural world continues to advance; moreover, carrying a high number of observations facilitated a more robust test of the PE algorithms' handling of large Jacobian matrices.

The parameterization scheme included relatively dense pilot point networks to increase parameter flexibility in the upper layers of the nearfield area and sparser pilot point networks or piecewise‐constant zone parameterization in the farfield and deeper layers below the area of interest. Parameters included horizontal hydraulic conductivity, anisotropy, specific yield, specific storage, horizontal conductance of four faults, zoned streambed vertical conductivity multipliers, zoned recharge multipliers by stress period, nearfield stream end inflow multipliers by stress period, and one multiplier applied to all pumping in all stress periods. The uppermost aquifer is of most interest to stakeholders in the area, thus parameter flexibility was focused on layers 1 and 2. This parameterization design resulted in 1777 adjustable parameters used for PE testing. Only subspace regularization (i.e., singular value decomposition, SVD) was used; Tikhonov regularization was not invoked because differences in PEST and PEST++ Tikhonov algorithms could obscure the differences among the PE algorithms that were the focus of our tests.

### Traditional Computationally Intensive PE Approach

A “traditional” approach to calibration was used based on guidelines for highly parameterized environmental models suggested by Doherty and Hunt (2010). Although Doherty and Hunt (2010) suggest the use of superparameters (Tonkin and Doherty 2005) to lessen the computational burden, here SVD was invoked without superparameters and each PE iteration required a full base‐parameter Jacobian be filled. This represents the most computationally intensive endmember (most forward runs needed) to complete the PE but ensures that the SVD is used on a Jacobian matrix most representative of each upgraded parameter set. Therefore, it reflects the most full representation of perturbation‐approximate sensitivity available, one less confounded by assumptions of sensitivity linearity over the range of parameters explored. Use of traditional SVD instead of superparameters also ensures that noise in the Jacobian matrix does not ripple to suboptimal superparameter definition, which then would be hardwired for the remainder of the PE process.

### Simultaneous Increments PE Approach

The simultaneous parameter increments approach to finite‐difference derivatives calculation varies more than one parameter during each forward model run used to fill the Jacobian matrix (Doherty 2020). Parameters included within a single forward run are those with unique (or almost unique) attribution of changes in model outputs. The most general workflow was used here, where a full Jacobian was constructed in the first PE iteration and then the algorithm sorted observations into groups that are sensitive to different groups of parameters, and those where cross‐sensitivities outside of these groupings are small. Simultaneous parameter increments can work well where the Jacobian matrix has a natural block structure. A block Jacobian, or quasi‐block Jacobian, can emerge from parameter to observation physical proximities; some parameters influence model outputs that are close to them in model space and time more than model outputs which are far from them. Under these circumstances, the simultaneous increments approach can take advantage of “localization” concepts that are used extensively in implementation of ensemble methods (e.g., Anderson 2007; Chen and Oliver 2017).

Although the simultaneous parameter increments method can work well in specific circumstances, it may encounter difficulties where blocks of parameter‐to‐model‐output influence are not clearly expressed. In these situations a modeler can define blocks manually in a heuristic manner, often based on parameter‐to‐observation distance. Alternatively, a quasi‐block structure can be attributed to a Jacobian matrix by assigning values of zero to low sensitivities that occupy many of the elements of a full finite‐difference Jacobian matrix. The former option has the disadvantage that distance is not the only factor that affects Jacobian matrix blockiness (e.g., a stream observation can be influenced by even distant up‐catchment model parameters). The latter option has the disadvantage of (1) numerical cost (requiring an initial full Jacobian matrix); (2) potential for inaccurate subdivision of discrete observation‐to‐parameter sensitivity blocks due to spurious sensitivities from numerical artifacts within the computer code, and (3) not accounting for PE nonlinearity that would cause the subdivision to vary during the PE process. For testing here, low sensitivities were assigned zero sensitivity; no manual definition of parameter‐observation blocks was performed.

### Iterative Ensemble Smoother PE Approach

The iterative ensemble smoother (IES) approach uses empirical correlations between an ensemble of parameter values and the resulting ensemble of simulated outputs to derive an approximate Jacobian matrix from a Monte Carlo‐style ensemble evaluation (Chen and Oliver 2013; White 2018; White et al. 2020). Rather than being a function of the number of adjustable parameters, the number of model runs to fill the approximate Jacobian matrix during each iteration is controlled by the number of user‐selected realizations in the parameter ensemble, where the number of realizations reflects the expected dimensionality of the solution space of the PE problem. The IES approximate Jacobian matrix is then used in a matrix/ensemble form of the Gauss‐Levenberg‐Marquardt algorithm to simultaneously adjust the full parameter ensemble (e.g., White 2018). In this way, the IES algorithm propagates an ensemble of multiple parameter sets through the PE process of adjusting parameters in response to residuals.

White et al. (2020) describe a potential disadvantage of the IES method: using an ensemble size that is much smaller than the number of parameters can result in spurious correlation. These nonphysical parameter‐to‐observation relations can then lead to “ensemble collapse,” where realizations are discarded and the posterior variance of the parameters is underestimated (e.g., Chen and Oliver 2017). Similar to the simultaneous increments PE approach, localization can avert ensemble collapse (e.g., Anderson 2007). Carrying a higher number of realizations can also off‐set adverse effects of realization loss, albeit at a higher computational cost. Typically, an ensemble size between 50 and 150 realizations is appropriate for most environmental modeling applications; here, however, 300 realizations were used to ensure the solution space was fully represented and results were free from adverse effects of ensemble collapse.

### Randomized Jacobian PE Approach

The randomized Jacobian approach to PE draws its inspiration from ensemble methods. It calculates a rank‐deficient approximation to a Jacobian matrix using random realizations of parameter increments, where increments can be on individual parameters or could be drawn from arbitrary prior probability distributions. Similar to IES, the number of these realizations need only be as large as the dimensionality of the solution space that is typically much smaller than the number of adjustable parameters (e.g., 300 realizations were used here). Unlike ensemble methods, however, it does not carry an ensemble of realizations or attempt to quantify post‐history‐matching parameter uncertainty. Rather, its focus is on efficient attainment of a single set of parameters representing the minimum error variance solution to a PE problem. In this way, the PE results are intended to be similar to those obtained using the traditional highly parameterized PE approach described by Moore and Doherty (2005), Hunt et al. (2007), and Doherty and Hunt (2010), among others.

Random realizations of parameter increments are performed to help increase the rank of the previously calculated Jacobian matrix; this combined Jacobian matrix is then used for parameter upgrade calculation during any particular iteration. The number of random increments on which Jacobian calculation is based can be varied upward on an as‐needed basis as the PE process progresses. Random parameter increments can be calculated based on assumptions of parameter independence, or on one or a number of user‐supplied covariance matrices, and can vary from iteration to iteration. Effects of spurious results and numerical noise can be addressed using options for autolocalization and flattening of the singular value spectrum (Doherty 2020). Similar to the implementation of the other PE approaches, these advanced options were not invoked for testing performed here.

## Results and Discussion

**Figure 5:**
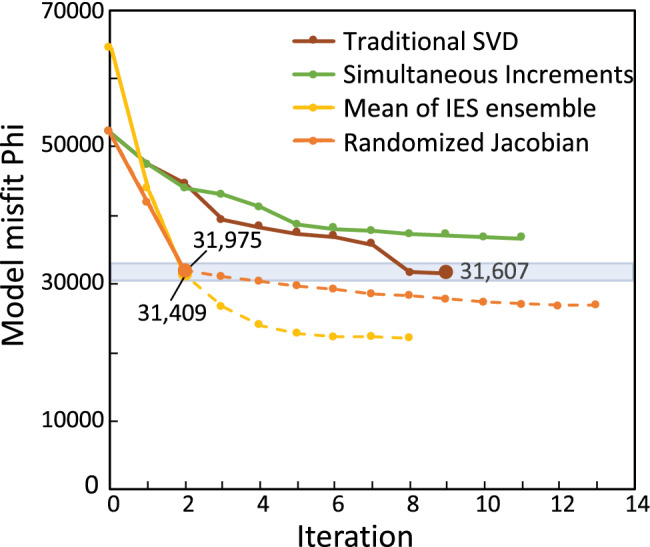
Change in model fit for the four approaches by PE iteration. The blue shaded rectangle represents a “desirable” range of fit, one reflecting adequate history matching and reasonable parameter fields. Larger symbols indicate iteration used for comparison; dashed lines reflect overfitting. IES, iterative ensemble smoother; phi, measurement objective function; SVD, singular value decomposition.

Three of the four PE approaches attained a desired fit, which was defined as a measurement objective function near 31,000—representing around a 39% reduction in the initial value misfit (Figure 5). This level of fit was deemed desirable because it exhibited an adequate history matching of the observations while maintaining reasonable parameter values (determined by stakeholders familiar with hydraulic properties in the area). Note that the IES PE approach has a higher starting misfit because it is reported as the mean of the ensemble of 300 IES realizations, which were drawn from the prior parameter distribution (i.e., stochastic parameter values) and observations included noise added during construction of the ensemble. Non‐IES approaches started at points in parameter space determined by initial coarse manual fitting to raw observations.

The simultaneous increments PE approach using default values did not attain the desired level of fit before the internal PE closure criteria were met and the PE process terminated (Figure 5). Recall that the PE closure criteria were the same for all four PE approaches, so the inability to reach the desired measurement objective function can be attributed to the PE approach. We interpret the lack of misfit reduction to the signal‐to‐noise ratio of the underlying Jacobian matrix being too low for the PE process to discern productive parameter upgrades. The propensity for low signal‐to‐noise ratios for our test problem is perhaps not surprising given the larger number of observations. However, the number of observations was similar for all PE approaches tested—why might this approach be most affected?

The poor performance is likely a result of a Jacobian matrix that does not exhibit sufficient blockiness when low sensitivities were assigned zero values—a suboptimal grouping that was likely compounded by numerical noise within the inverse problem. Yet a modest improvement to fit was observed in early PE iterations using the simultaneous increments approach because noise and suboptimal parameter block selection were not sufficiently large to hide the broad relations between the parameters and observation dataset evaluated during the PE process. Recall that SVD of the Jacobian matrix (a numerical device, i.e., fundamental to all of the PE approaches discussed here) defines combinations of parameters based on the broadscale system response expressed in the observations. To achieve an improved fit with the calibration dataset in the early stages of an inversion process, these combinations do not need to be optimal; they only need to contain enough information to construct an upgrade to the parameters that results in a better fit (i.e., have a nonzero projection onto the solution space of the inverse problem).

After some modest improvement to fit, the use of simultaneous increments approach stalls because the combinations of parameters that can be adjusted to improve model‐to‐measurement fit are implicitly hardwired into the assumed block structure of the Jacobian matrix. If this blocky structure is suboptimally defined, the effectiveness of any calculable parameter upgrade will be limited. However, even if it is optimally defined for a high‐quality Jacobian matrix, it may be inappropriately defined for a poor‐quality Jacobian matrix that is contaminated by numerical noise. In such a context, the ability of simultaneous increment's implicitly hardwired combinations of parameters to “do the job” is inferior to that of combinations of random parameters or ensemble of realizations that can more readily adapt to the nuances of a Jacobian matrix evolution that occurs as PE progresses.

Or put another way, the simultaneous increments approach can be thought of another form of localization, where meaningful parameter‐observation relations can be lost if localization relations are mis‐specified, which results in a lesser fit. Recall too, not all parameter‐observation sets have the same ability to inform the PE process. Rather, the nonlinear regression is primarily driven by influential observations (e.g., Yager 2004; Hunt et al. 2006). As a result, quality sensitivity estimates can overcome noisy, inaccurate estimates if enough quality estimates are available for observations influential for the regression. Simplifications that give computational efficiency but do not provide a good approximation of sensitivities related to influential observations cannot reach the same performance as those that better represent salient information within the Jacobian matrix. The simultaneous increments approach drops parameters from the Jacobian calculations for efficiency without evaluating observation influence, and was not sufficiently flexible to improve its simplification scheme as the PE progresses.

**Figure 6:**
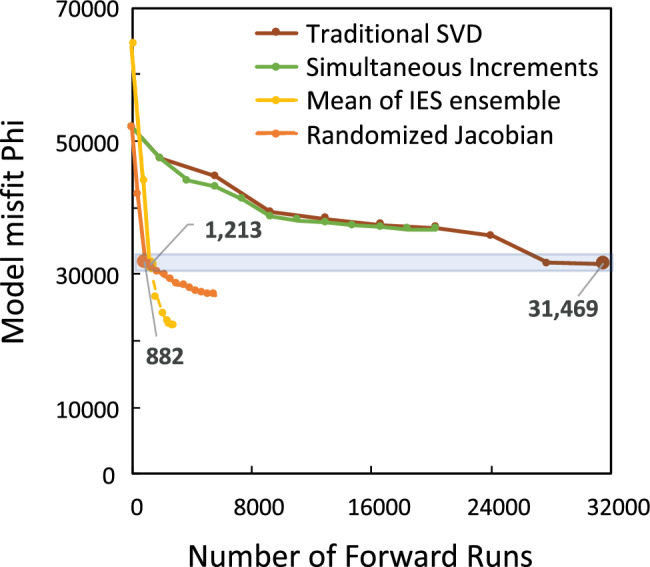
Improvement of fit versus number of forward model runs of the MODFLOW‐NWT model (>6 h per run). The blue shaded rectangle represents a “desirable” range of fit, one reflecting adequate history matching and reasonable parameter fields. Larger symbols indicate iteration used for comparison; dashed lines reflect overfitting. IES, iterative ensemble smoother; phi, measurement objective function; SVD, singular value decomposition.

Of the three PE approaches that reached the desired level of fit, the traditional SVD approach took many more runs to attain the fit (Figure 6), and only attained the fit near the end of the PE process. Why did this PE approach take more PE iterations and end at a misfit higher than the other successful methods? We again attribute this to the signal‐to‐noise ratio in the Jacobian matrix. Recall that in the traditional approach the full Jacobian matrix is filled each PE iteration. This full matrix has all the signal for all observations influential to the regression, but also all of the noise of those that are not. This larger noise component reduces the efficiency of the parameter upgrade selection. Indeed, the ultimate best fit of the traditional method appears to be only coincidentally meeting the desired fit, and may not have if not for a fortuitous choice in parameter upgrades between PE iteration 7 and 8 (Figure 5). Though not tested here, the traditional SVD approach may have attained lower measurement objective functions (phis) in fewer PE iterations if it used a larger singular value truncation threshold (thus retaining fewer singular values in the solution). Moore and Doherty (2006) show how this threshold can off‐set the adverse effects of spurious parameter values when the PE process has high amounts of measurement or numerical noise. However, the number of forward runs is expected to still be appreciably higher even with such a modification.

Both the randomized Jacobian and IES PE approaches reached the desired level of fit very quickly (two PE iterations) and in appreciably fewer runs than the traditional approach—in fact, fewer than the number of parameters in the PE problem. Given the >6 h runtime of the forward run and the large number of parameters, this reduction in total runs translates to a large reduction in wall‐clock time needed. Although both the randomized Jacobian and IES approaches found lower misfits than the traditional approach, the larger exploration of the parameter space by the IES ensemble found appreciably lower measurement objective functions after PE iteration 2 (Figure 5). For our test problem, however, values below 31,000 were found to contain extreme parameter values and thus were deemed unacceptably overfit. This finding is consistent with the experience of the authors that results of the randomized Jacobian and IES approaches typically have an increase in extreme parameter values after PE iteration 2 or 3.

The high level of efficiency for these two methods indicates that they can capture the signal that is resident in influential observations while allowing augmented exploration of parameter space beyond what the localized Jacobian typically performs. Consider noise in gradients calculated from the Jacobian matrix: by using gradients derived from sampling across parameter space, the effect of noise in a local gradient approximation (such as that arising from solver tolerance selection—Figure 1) is mitigated. Noise in the solution process is mitigated by smaller fundamental subspaces compared to a full Jacobian matrix. Recall that the Jacobian matrix is the means to an end, where the end is a better update to the parameters—an approximation to the Jacobian will not hurt the PE process if it lowers the noise floor and amplifies the signal for influential observations. Approaches based on random parameter increments or random IES realizations provide flexibility of parameter upgrade definition capable of capturing the information that is contained in broadscale system behavior, overcoming measurement and numerical noise that contaminates this information. This, in turn, allows very efficient discernment of improved parameter sets in early iterations of a PE process.

The Jacobian matrix used in both the IES and randomized Jacobian approaches is formed from empirical cross‐covariance between parameters and observations. However, in IES the parameter increments used to construct the Jacobian matrix are based on an ensemble of parameters and observations rather than local parameter perturbation. Therefore, in addition to the benefits of randomness, the IES ensembles explore larger portions of the parameter space during each PE iteration. Local minima to the objective function can occur in highly nonlinear PE problems or when model outputs are contaminated by numerical noise, as was the case in our test model. The enhanced exploration helps IES overcome local minima and reach a lower measurement objective function than approaches using local perturbation sensitivity methods.

Finally, the IES approach implicitly amplifies the signal‐to‐noise ratio by focusing on an implied lower‐dimensional subspace (as expressed by a relatively low number of realizations in the ensemble compared to the number of parameters) within the much larger model output space. Mathematically, this means that the IES algorithm tends to reduce the magnitude of the primary eigencomponents of the residual matrix. Conceptually, this implies that the broad, regional residual patterns in space and in time are reduced, which facilitates utilizing information that informs smaller‐scale system detail. However, simulating observations that depend on system detail will often result in more extreme parameter values. Lower measurement objective functions in later PE iterations, therefore, are expected to be more prone to overfitting and thus less desirable. As a result, for the applied modeling context, the ability to quickly attain a desired level of fit in early PE iterations is likely a more important metric than the ultimate magnitude of the objective function.

**Figure 7:**
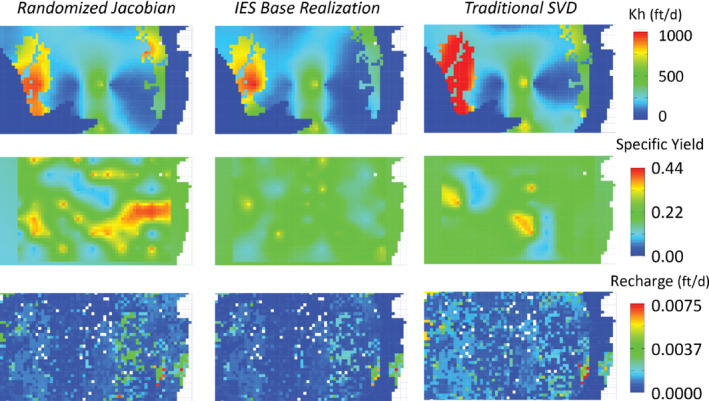
Optimal parameters for the nearfield area of interest at the desirable level of fit, where the IES results reflect a single parameter field from the PEST++ reported “base” realization. The location of the nearfield is shown in Figures 3 and 4. IES = iterative ensemble smoother; Kh = horizontal hydraulic conductivity; specific yield [dimensionless]; SVD = singular value decomposition.

The agreement of the resulting parameter sets identified among the PE approaches was generally good. Although both layer 1 and 2 reflect the shallow system most of interest to stakeholders, layer 1 had an appreciable area of desaturation due to pumping. Therefore, the more complete parameter distributions in layer 2 are used for comparison. Horizontal hydraulic conductivity and recharge results were similar among the methods (top and bottom rows of Figure 7). The specific yield results appear to have less agreement (middle row of Figure 7); however, the plot coloring accentuates differences and the range of optimal parameters depicted at the color extremes is small (0.1 to 0.4). Moreover, no additional regularization (e.g., Tikhonov regularization) was used in this test problem to emphasize the differences within the PE solution algorithms themselves.

Perfect agreement among PE approaches is not expected for most environmental modeling problems due to: (1) inherent nonuniqueness of the inverse problem for environmental systems; (2) different paths through solution space taken as the Jacobian matrices differ among the PE approaches; and (3) the subjective selection of a desirable result rather than a quantitative best‐fit selected by an algorithm. Furthermore, this particular modeling problem is highly dependent on surface water flows generated from outside the nearfield model domain (which are specified as parameters in the PE); modeling problems that generate more of the important sources of water within the model domain might be expected to exhibit less variability among different methods.

## Conclusions and Suggestions for Applied Modeling

The work presented here demonstrates large PE efficiencies can be gained by advanced PE algorithms, even on complex, realistic environmental models with many observations and parameters. All advanced methods reduced the forward runs needed for PE; however, the simultaneous increments were not able to obtain the desirable fit of the other methods using the default values chosen. For the test problem, the traditional SVD approach described by Doherty and Hunt (2010) was able to meet the desired level of fit but the number of forward model runs was over 25 times that of the more efficient methods, and the resulting parameters contained more extreme values. The randomized Jacobian and IES PE methods that performed most efficiently provided reasonable parameter sets with relative few forward model runs—indeed, the desirable fit was found in less total forward runs than the number of parameters included in the PE problem. Thus, for this problem, either the randomized Jacobian or IES PE approach would provide a result much faster than traditional methods. Such quick turnaround of PE insight facilitates additional PE trials more often during the course of effort, which in turn should result in more insight into the problem, and more opportunity for uncertainty assessment and management optimization, given the same modeling budget.

Results of the randomized Jacobian approach are consistent with the well‐established PEST single minimum error variance solution, which is typically characterized as having smooth parameter fields (Moore and Doherty 2005). The established procedure to access PEST's Tikhonov regularization to reign in overfitting (e.g., Doherty and Hunt 2010) and obtain a minimum error variance parameter field is therefore a strength of the approach. A single minimum error variance solution, as well as familiar output file formats, facilitate a seamless transition for traditional PEST users. However, like established PEST, uncertainty assessment requires additional processing after calibration. The strength of IES, on the other hand, is that uncertainty assessment is coproduced during calibration, and the uncertainty characterized in the ensemble is likely to better characterize the endmember forecasts. In addition, IES methods are well suited to handle local minima and very nonlinear problems. However, the IES approach requires a shift to an ensemble mindset, where statistics describe the PE results rather than a single minimum error variance solution. In recognition of this potential issue, IES methods in PEST++ include a “base” realization that retains many of the features of the initial parameter values during IES realization generalizations; the base realization can be tracked separately by the modeler as the calibration progresses. But in practice, the PEST++ base realization run does not exactly equate to a PEST minimum error variance result. PEST performs several mitigation measures to account for the bias inherent to a rank‐deficient Jacobian matrix. However, PEST++ does not; thus, its base realization generally has different parameter fields (such as seen in Figure 7).

Yet focusing on an “either‐or” between the PE approaches may miss an important opportunity: many modeling problems may benefit from use of both approaches, whereby the randomized Jacobian is used for initial calibration and IES is subsequently used to quantify uncertainty. That is, using a quickly calibrated randomized Jacobian minimum‐error variance result to generate IES realizations provides a more representative initial view of the system, which would facilitate higher quality realizations in the ensemble used for uncertainty analysis. Evaluating a combined approach is a topic for future work.

## Authors' Note

The authors do not have any conflicts of interest or financial disclosures to report.

## Supporting information


**Appendix S1.** Description of MODFLOW‐NWT model used for evaluating lower computational burden approaches for calibration of large environmental models.
**Figure S1.** Location of the model domain, Mississippi Delta, nearfield and composite hydrograph areas.
**Figure S2.** Parameter zones for each model layer.
**Figure S3.** Simulated major water budget components by stress period in the Mississippi Delta area
