# Supplementary material for: Evaluating Lower Computational Burden Approaches for Calibration of Large Environmental Models
Source: Ground Water. 2021 Jun 8;59(6):788–98. doi: 10.1111/gwat.13106 (PMC9292030; doi:10.1111/gwat.13106)
Supplement: Supplementary file 1 — Appendix S1. Description of MODFLOW‐NWT model used for evaluating lower computational burden approaches for calibration of large environmental models. Figure S1. Location of the model domain, Mississippi Delta, nearfield and composite hydrograph areas. Figure S2. Parameter zones for each model layer. Figure S3. Simulated major water budget components by stress period in the Mississippi Delta area [file GWAT-59-788-s001.docx]

**Supporting Information Appendix S1**

**Description of MODFLOW-NWT model used for “Evaluating Lower Computational Burden Approaches for Calibration of Large Environmental Models”**

Randall J. Hunt^1^, Jeremy T. White^2^, Leslie Duncan^3^, Connor J. Haugh^3^ and John Doherty^4^

^1^U.S. Geological Survey, Upper Midwest Water Science Center, Middleton, WI USA (corresponding author) [rjhunt@usgs.gov](mailto:rjhunt@usgs.gov)

^2^Interra, Inc. Fort Collins, CO USA.

^3.^U.S Geological Survey, Lower Mississippi Gulf Water Science Center, Nashville, TN USA

^4^Watermark Numerical Computing, Brisbane, Queensland, Australia

**Abstract from Associated Research Paper**

Realistic environmental models used for decision making typically require a highly parameterized approach. Calibration of such models is computationally intensive because widely used parameter estimation approaches require individual forward runs for each parameter adjusted. These runs construct a parameter-to-observation sensitivity, or Jacobian, matrix used to develop candidate parameter upgrades. Parameter estimation algorithms are also commonly adversely affected by numerical noise in the calculated sensitivities within the Jacobian matrix, which can result in unnecessary parameter estimation iterations and less model-to-measurement fit. Ideally, approaches to reduce the computational burden of parameter estimation will also increase the signal-to-noise ratio related to observations influential to the parameter estimation even as the number of forward runs decrease. In this work a Simultaneous Increments, an iterative ensemble smoother (IES), and a Randomized Jacobian approach were compared to a traditional approach that uses a full Jacobian matrix. All approaches were applied to the same model developed for decision making in the Mississippi Alluvial Plain, USA. Both the IES and Randomized Jacobian approach achieved a desirable fit and similar parameter fields in many fewer forward runs than the traditional approach; in both cases the fit was obtained in fewer runs than the number of adjustable parameters. The Simultaneous Increments approach did not perform as well as the other methods due to inability to overcome suboptimal dropping of parameter sensitivities. This work indicates that use of highly efficient algorithms can greatly speed parameter estimation, which in turn increases calibration vetting and utility of realistic models used for decision making.

**Introduction**

The groundwater model used for the analysis presented represents an update to the existing groundwater model of Haugh et al. (2020a,b). Haugh et al. (2020a) itself is an update to earlier efforts to simulate the Mississippi Embayment regional aquifer system (MERAS). The original MERAS model was a MODFLOW-2005 (Harbaugh 2005) model documented by Clark and Hart (2009), which was subsequently updated by Clark et al. (2011) and Clark et al. (2013). Model input and output for our work can be accessed via an online model archive (Hunt et al. 2021). It uses MODFLOW-NWT (Niswonger et al. 2011) and leverages products recently developed for the U.S. Geological Survey (USGS) Mississippi Alluvial Plain (MAP) project. The MAP effort is planned to culminate with a state-of-the-art MODFLOW6 regional and inset models; thus, the MODFLOW-NWT model used here represents an interim precursor to final MAP products. The purpose of the current work is to illustrate the calibration approaches that form the thrust of our presentation and serve as an interim tool for water management scenario analysis.

The overarching model design, grid discretization, boundary conditions, and limitations and assumptions for the MODFLOW-NWT model used in this publication are found in the source references of Clark and Hart (2009) and Clark et al. (2011; 2013). In this Supplemental Information section, we list the changes to the current published MODFLOW model and provide references for the associated data sources. The organization focuses on what is not documented in MODFLOW-2005 models already published. An online model archive is also available (Hunt et al. 2021).

**Updates to Existing Models**

The original MERAS models were developed and calibrated using data from the larger 7-state system; recent water management scenarios run with the model (e.g., Haugh et al. 2020b), however, typically focused on the Mississippi Delta (Haugh et al. 2020a). Given our objective to test calibration methods that would facilitate scenario forecasts, our MODFLOW-NWT formulation simulated the larger model domain of Clark and Hart (2009) but focused the parameterization flexibility and calibration effort on the center of Mississippi Delta area of the MERAS model domain (called the “nearfield” – figure S1-1).

**Model Code, Packages, and Temporal Discretization**

The MODFLOW-2005 (Harbaugh 2005) input was moved to MODFLOW-NWT (Niswonger and others 2011). A substantive change was changing the previous conceptualization of a convertible top layer and confined layers 2 through 13 needed for stability in the MODFLOW-2005 solution to convertible layers for all 13 layers. This allowed both Specific Yield (Sy) and Specific Storage (Ss) to be entered for each layer. Advantages that MODFLOW-NWT can provide, such as this enhanced simulation capability, have been discussed by Hunt and Feinstein (2012).

The simulation time was also reduced to shorten the forward runtime for our testing. The most recent 83 stress period MODFLOW-2005 model (Haugh et al. 2020) was divided into two sequential MODFLOW-NWT runs. The first represented a 10-year/20 stress period simulation reflecting dynamic equilibrium initial conditions (pg. 312 in Anderson et al. 2015) ending 30 September 2008. The initial conditions for the dynamic equilibrium run were obtained from a pre-development steady state simulation of the system. The dynamic equilibrium initial-conditions were piped to the second 15-stress period MODFLOW-NWT run as starting heads. The second MODFLOW-NWT model represented a 7.5-year/15 stress period simulation, where the 15 stress periods had 7 stress periods of spin up (pg. 313 in Anderson et al. 2015), and 8 stress periods of history matching. The spin-up/calibration run simulated conditions from 1 October 2008 to 31 March 2016. For both MODFLOW-NWT models, stress periods were 0.5 year long and aligned with growing (summer) and non-growing (winter) seasons.

**Recharge**

The USGS MAP Project has developed new representations of groundwater recharge using the Soil-Water-Balance (SWB) code of Westenbroek et al. (2017). The methodology and MAP source data sets for the 15 stress periods used in MODFLOW-NWT modeling are reported in Westenbroek et al. (2021a,b,c). Recharge was parameterized using the same zones and multipliers from the previous MERAS models as first documented in Clark and Hart (2009).

**Surface-water Representation**

The USGS MAP Project has developed more expansive representations of the stream network using the utilities of Leaf et al. (2021). The methods create Stream-Flow Routing (SFR) Package input files; these MAP-related elements are also documented in Leaf et al. (2021). Initial estimates of streambed vertical hydraulic conductivity were obtained from MAP water-borne geophysics efforts (Miller et al. 2016, Miller et al. 2018, Adams et al. 2019). To minimize adverse effects of model structure error from non-calibrated areas outside the nearfield, end-inflows were specified to the SFR input at the edge of the Mississippi Delta nearfield area based on MAP estimates of surface-water flow in these areas using the information contained in Dietsch et al. (2021) and Westenbroek et al. (2021d,e). The resulting SFR network was appreciably denser than previous MERAS models (figure 2 in corresponding main text publication).

**Pumping**

Average 1998-2007 pumping rates were used to represent pumping rates of the dynamic cyclic equilibrium model used to construct initial conditions. The pumping rates were derived from the rates given in Clark et al. (2009). Pumping rates for the 15-stress period model for withdrawals from the alluvial aquifer were obtained from the MAP water use estimates using the model of Wilson (2020). Rates given in Clark et al. (2009) were used for withdrawals from the deeper aquifers below the shallow aquifer that was the focus of the study.

**Observations used for History Matching**

Observations for history matching were specified for all 15 stress periods for evaluation, but observations from the spin-up 7 stress periods were not weighted for calibration and therefore only qualitatively evaluated; only observations in the last 8 stress periods were weighted for history matching. Observations were developed using both USGS measured data and processed hydraulic heads and streamflows from Asquith et al. (2019; 2020) and Dietsch et al. (2021), respectively. For instances where multiple measured water levels were present within a stress period, the average of the measured water levels was used as the hydraulic head observation. The history matching focused on nearfield hydraulic heads and fluxes (figure S1; figure 4 in corresponding main text publication). The averaging inherent to the 6-month stress period length precluded extensive use of temporal difference targets (pg. 382, Anderson et al. 2015). Therefore, hydraulic head and flux difference targets throughout the model domain were evaluated, but only select locations in the nearfield were given weight for history matching to account for their importance for scenario forecasts. Hydraulic head and flux targets for areas outside the nearfield were also qualitatively evaluated but not weighted for history matching.


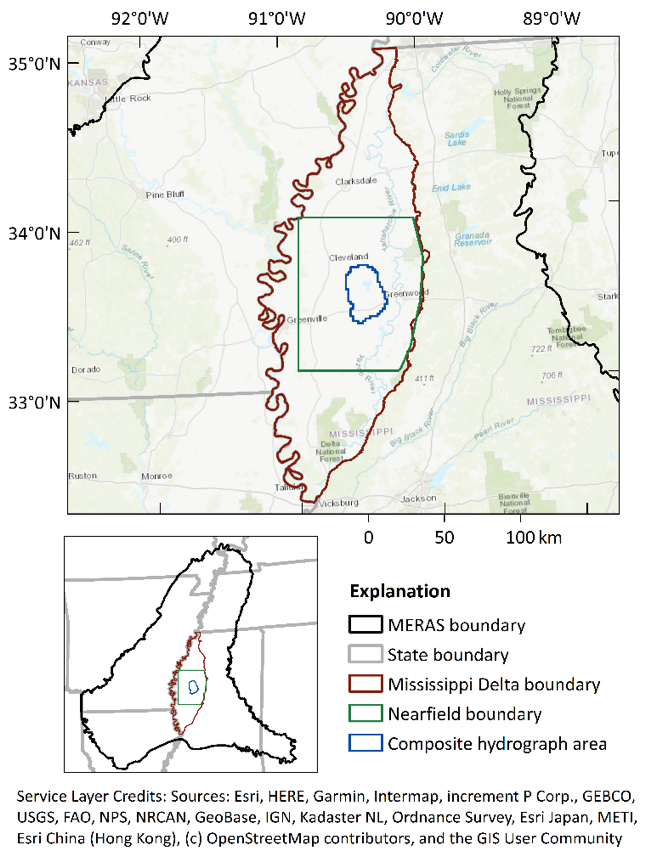


*Figure S1. Location of the model domain, Mississippi Delta, nearfield and composite hydrograph areas.*

**Parameterization**

Parameterization used a highly parameterized approach of Hunt et al. (2007) using the guidelines of Doherty and Hunt (2010). Pilot points (e.g., Doherty 2003, Doherty et al. 2010) were used to represent horizontal hydraulic conductivity (layers 1-13), anisotropy (layers 1-13), and Specific Yield (Sy) (layers 1-2). Given the focus on shallow high-capacity pumping, parameter flexibility focused on the upper two layers in the Mississippi Delta nearfield model area, as noted by a denser pilot point network (figure 3 in corresponding main text publication). Less-dense pilot point networks for these model inputs were used outside of the nearfield. Pilot point values were estimated, and a zone multiplier was also applied using the approach of McKenna et al. (2019). The zones were derived using the aquifer and confining unit zones of Clark and Hart (2009); modeled areas without pilot points were simulated using the same zones as piecewise constant zone parameters (figure S2).

One multiplier was applied to the entire recharge array for the dynamic-equilibrium MODFLOW-NWT run used to develop the initial conditions for the 15-stress period simulation. For the 15-stress period simulation, multipliers were applied to the SWB recharge arrays using the recharge zones of Clark and Hart (2009); each individual stress period had multipliers derived from the Clark and Hart (2009) zones. Five multipliers were applied to streambed vertical hydraulic conductivity (Kv) – the multipliers were applied to the following groups of initial estimates of Kv: less than or equal to 1.0 (245 cells), 1.0-1.3 (10505 cells), 1.3-2.6 (5463 cells), 2.6-5.2 (4393 cells), and greater than or equal to 5.2 ft/day (5029 cells). A single multiplier was used to adjust all wells in the MODFLOW WEL Package input, where one multiplier was applied to all 15 stress periods. One parameter per layer was used for all Specific Storage input, and for Sy in layers 3 through 13.


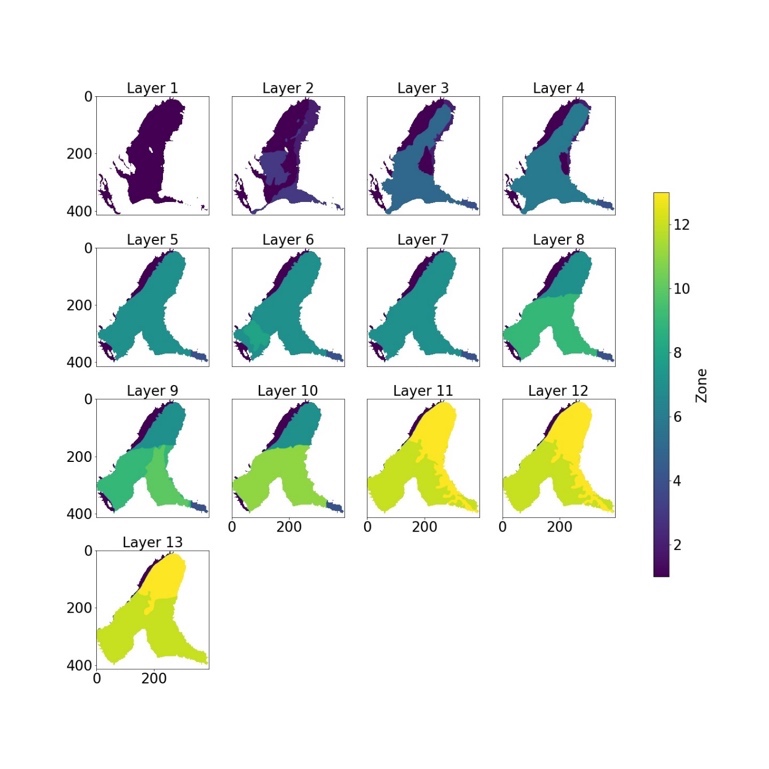


*Figure S2. Parameter zones for each model layer.*

**Simultaneous Increments Parameter Estimation Settings**

For Simultaneous Increment runs, the following input was added to the PEST control (*.pst) file after the “singular value decomposition” section; text after the # is optional and is included here to identify the PEST variables contained on each line. These variables apply to PEST_HP Version 16.1.

* simultaneous parameter increments

0 0 #BLANKJCO SIMINC

blanking_file = #BLANKFILE

si_file = #SIFILE

1 #SIMINCALC

.05 .05 999 0 100 1 #FRACOBS FRACPAR FULLJCOITN SIMINCACCEL MINSIMINC MATCHAGENTS

The PEST_HP manual defines each control variable in detail; thus, they are only briefly described here.

BLANKJCO is an integer variable that indicates whether a user specified Jacobian matrix blanking file is used. A value of 0 indicates that no user-specified blanking file is provided.

SIMINC is an integer variable that instructs PEST_HP to use a simultaneous parameter increment strategy from a user-specified simultaneous increment file.

BLANKFILE is the filename of the user-specified blanking file.

SIFILE is the filename of the user-specified simultaneous increment file.

SIMINCCALC is an integer variable that informs PEST_HP whether it must periodically calculate a blanking matrix and devise a simultaneous parameter increment strategy itself. A value of 1 invokes these capabilities.

FRACOBS and FRACPAR: When invoked, PEST_HP calculates a blanking matrix from a Jacobian matrix by blanking all rows of the Jacobian matrix for which observation weights are zero. Then, for each row, it ascertains the weighted sensitivity of highest absolute value. Any elements within this row whose absolute weighted sensitivities are less than FRACOBS times this maximum value are then blanked. The same operation is then performed for each column of the weighted Jacobian matrix using FRACPAR as the governing variable. FRACOBS and FRACPAR are real variable between 1.0 and 0.0.

FULLJCOITN specifies the parameter estimation iteration interval for when a full Jacobian matrix is calculated using the standard finite-difference methods. When set to 999, no full Jacobian matrices will be calculated after the first iteration.

SIMINCACCEL for “simultaneous increment acceleration” informs PEST_HP whether it should update its blanking matrix or simultaneous parameter increment strategy. The value 0 means that no updates to the blanking matrix or simultaneous parameter increment strategy will occur.

MINSIMINC is the minimum number of simultaneous parameter increments.

MATCHAGENTS set to 1 tells PEST_HP to ensure that the number of simultaneous parameter increments that it adopts is an integral multiple of the number of agents available for carrying out model runs.

**IES Parameter Estimation Settings**

For the IES results shown, the following PEST++ Version 5 control variables were appended as separate lines at the end of the PEST control file.

++par_sigma_range(6.0)

++ies_initial_lambda(10000)

++ies_lambda_mults(0.01,0.1,1,10,100)

++lambda_scale_fac(0.1,0.25,0.75,1.0,1.1)

++ies_num_reals(300)

++ies_subset_size(8)

++ies_no_noise(true)

++ies_drop_conflicts(false)

White et al. (2020) and the PEST++ user manual describe each variable in detail; brief descriptions are provided below.

++par_sigma_range(): The difference between a parameter’s upper and lower bounds expressed as number of standard deviations.

++ies_initial_lambda() is the initial Marquardt lambda.

++ies_lambda_mlts(): factors by which to multiply the best lambda from the previous iteration to yield values for testing parameter upgrades during the current iteration.

++ies_lambda_scale_fac(): Line search factors along parameter upgrade directions computed using different Marquardt lambdas.

++ies_num_reals(): The number of realizations to draw in order to form parameter and observation ensembles.

++ies_subset_size(): Number of realizations used in testing and evaluation of different Marquardt lambdas.

++ies_no_noise(): Flag to not generate and use realizations of measurement noise.

++ies_drop_conflicts(): Flag to remove nonzero weighted observations that are in a prior-data conflict state from the upgrade calculations.

**Randomized Jacobian Parameter Estimation Settings**

For the Randomized Jacobian runs, the following input was added to the PEST control (*.pst) file after the “singular value decomposition* section; text after the # is optional and is included here to identify the PEST variables contained on each line. These variables apply to PEST_HP Version 16.1.

* randomized jacobian

1 222 #RANDOMJAC RANDOMSEED

300 0 300 #NRANDOMSTART NRANDOMINC NRANDOMFIN

2.0 4 1 #RANDINCFAC NRANDREPEAT EMPIRICSTD

0.3 #RANDJACRETAIN

0 0.1 4 5 #SWITCH_TO_FD PHIREDFD NOPTSWITCHFD REPEATTOL

The PEST_HP manual defines each control variable in detail; thus, they are only briefly described here.

RANDOMJAC: If set to 1, then Randomized Jacobian calculation is enabled.

RANDOMSEED is the seed for the random number generator.

NRANDOMSTART, NRANDOMINC, NRANDOMFIN: NRANDOMSTART is the initial value of number of random parameter sets used to build a diminished rank approximation to the Jacobian matrix. NRANDOMINC is the user specified increment applied to NRANDOMSTART if the value of the objective function is at or above the lowest value that has been achieved. NRANDOMFIN is the maximum number of random parameter sets.

RANDINCFAC is the parameter increment assigned to represent derivatives computed using two-point finite parameter differences.

NRANDREPEAT is the number of iterations existing parameter values are retained for the next iteration of the inversion to improve the quality of the covariance matrix.

EMPIRICSTD stands for “empirical standard deviation,” where 0 invokes theoretical, increment-derived parameter standard deviations to calculate the increment; 1 calculates parameter standard deviations empirically from the random values generated for respective parameters.

RANDJACRETAIN governs how much of the covariance matrix calculated during the previous iteration of the inversion process is retained in calculating the covariance matrix for the current iteration.

SWITCH_TO_FD, PHIREDFD, NOPTSWITCHFD and REPEATTOL: If, during a particular iteration of an inversion process based on Randomized Jacobian matrices, the objective function does not fall by a relative amount of PHIREDFD, then PEST_HP can switch to finite difference calculation of the Jacobian matrix. This occurs if the integer variable SWITCH_TO_FD is set to 1; a setting of 0 prevents this occurrence. The integer variable NOPTSWITCHFD specifies the first parameter estimation iteration that can switch to computationally costly finite-difference derivatives calculation. PEST_HP does not make the switch to finite-difference derivatives calculation until REPEATTOL iterations of covariance matrix enhancement have elapsed.

**Simulation Results**

Simulated major water budget components of the alluvial aquifer include groundwater withdrawals by wells, recharge from precipitation, exchange of water with streams, and storage gains and losses (figure S3). In the Mississippi Delta area, seasonal groundwater withdrawals by wells are a substantial outflow. The largest source of water to the alluvial aquifer is recharge from precipitation followed by streamflow losses into the groundwater system. Groundwater storage in the alluvial aquifer changes seasonally with water being removed from storage to help supply water to wells during the growing (summer) stress periods and water being replenish from the active groundwater flow system into storage during the non-growing (winter) stress periods (figure S3).


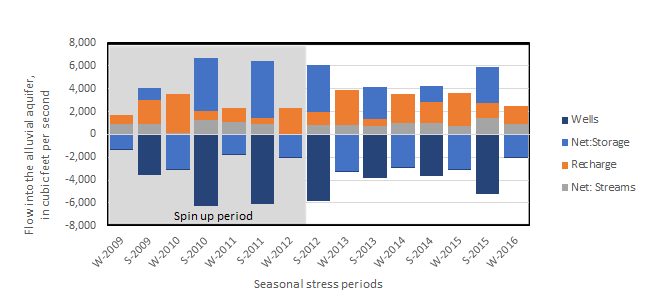


*Figure S3. Simulated major water budget components by stress period in the Mississippi Delta area*

**Limitations and Assumptions**

The objectives of this modeling effort were to (1) test new calibration approaches for large environmental models; and (2) evaluate new MAP data products with the quantitative framework of a groundwater model. Moreover, a subsequent MAP MODFLOW6 model represents the operational model needed for meeting decision support objectives of the MAP effort. Improvements to the system simulation were realized in the model documented here, including simulating all 13 layers as convertible layers which allowed a better representation of the aquifer storage properties as both Specific Yield (Sy) and Specific Storage (Ss) were calibrated independently. Additionally, the more detailed representation of the stream network allowed more refined simulation of groundwater and surface-water interactions. However, given its provenance, this most recent model has inherited a number of limitations as described by Clark and Hart (2009), Clark et al. (2013), and Haugh et al. (2020a), which can be addressed in future modeling. Therefore, although the model described here represents an improvement to prior MERAS groundwater models, it should be viewed as an interim representation of the MAP system built for testing new MAP data products and calibration approaches. Any use of trade, firm, or product names is for descriptive purposes only and does not imply endorsement by the U.S. Government.

**References**

Adams, R.F., B.V. Miller and W. H. Kress. 2019. Waterborne Resistivity Inverted Models, Mississippi Alluvial Plain, 2016-2018: U.S. Geological Survey data release. <https://doi.org/10.5066/P9WQPRFB>.

Anderson, M.P., W.W. Woessner, and R.J. Hunt. 2015. Applied Groundwater Modeling: Simulation of Flow and Advective Transport (2nd edition). Academic Press, Inc. 564 p. ISBN 9780120581030.

Asquith, W.H., R.C. Seanor, V.L. McGuire and W.H. Kress. 2019. Source code in R to quality assure, plot, summarize, interpolate, and extend groundwater-level information, visGWDB—Groundwater-level informatics with demonstration for the Mississippi River Valley alluvial aquifer: U.S. Geological Survey software release, Reston, Va. <https://doi.org/10.5066/P9W004O6>.

Asquith, W.H., R.C. Seanor, V.L. McGuire and W.H. Kress. 2020. Methods to quality assure, plot, summarize, interpolate, and extend groundwater-level information—Examples for the Mississippi River Valley alluvial aquifer. *Environmental Modelling and Software* 134: 104758, <https://doi.org/10.1016/j.envsoft.2020.104758>.

Clark, B.R., and R.M. Hart. 2009. The Mississippi Embayment Regional Aquifer Study (MERAS)—Documentation of a groundwater-flow model constructed to assess water availability in the Mississippi embayment: U.S. Geological Survey Scientific Investigations Report 2009–5172, 61 p. <https://pubs.usgs.gov/sir/2009/5172/>.

Clark, B.R., R.M. Hart and J.J. Gurdak., 2011. Groundwater availability of the Mississippi embayment: U.S. Geological Survey Professional Paper 1785, 62 p. <https://pubs.usgs.gov/pp/1785/>.

Clark, B.R., D.A. Westerman and D.T. Fugitt. 2013. Enhancements to the Mississippi Embayment Regional Aquifer Study (MERAS) groundwater-flow model and simulations of sustainable water-level scenarios: U.S. Geological Survey Scientific Investigations Report 2013–5161, 29 p. <https://pubs.usgs.gov/sir/2013/5161/>.

Dietsch, B. J., W.H. Asquith and S.M. Westenbroek. 2021. Estimation of Monthly Mean and Monthly Base Flow of Streamflow using Random Forests for the Mississippi River Alluvial Plain. USGS written communication.

Doherty, J. 2003. Ground water model calibration using pilot points and regularization. *Groundwater* 41, no. 2: 170-177. <https://doi.org/10.1111/j.1745-6584.2003.tb02580.x>.

Doherty, J., M.N. Fienen, and R.J. Hunt. 2010. Approaches to highly parameterized inversion: Pilot-point theory, guidelines, and research directions: USGS Scientific Investigations Report 2010–5168, 36 p. <https://pubs.usgs.gov/sir/2010/5168/>.

Doherty, J., and R.J. Hunt. 2010. Approaches to Highly Parameterized Inversion: A Guide to Using PEST for Groundwater-Model Calibration. USGS Scientific Investigations Report 2010–5169, 59 p. <https://pubs.usgs.gov/sir/2010/5169/>.

Harbaugh, A.W. 2005. MODFLOW-2005, the U.S. Geological Survey modular ground-water model -- the Ground-Water Flow Process: U.S. Geological Survey Techniques and Methods 6-A16.

Haugh, C.J., C.D. Killian and J.R.B. Barlow. 2020a. Simulation of water-management scenarios for the Mississippi Delta: U.S. Geological Survey Scientific Investigations Report 2019–5116, 15 p. <https://doi.org/10.3133/sir20195116>.

Haugh, C.J., C.D. Killian and J.R.B. Barlow. 2020b. MODFLOW-2005 model used to evaluate water-management scenarios for the Mississippi Delta: U.S. Geological Survey data release. <https://doi.org/10.5066/P9906VM5>.

Hunt, R.J., J. Doherty and M.J. Tonkin. 2007. Are models too simple? Arguments for increased parameterization. *Groundwater* 45, no. 3: 254-263. <https://doi.org/10.1111/j.1745-6584.2007.00316.x>.

Hunt, R.J., and D.T. Feinstein. 2012. MODFLOW-NWT – Robust handling of dry cells using a Newton Formulation of MODFLOW-2005. *Groundwater* 50, no. 5: 659-663. <https://doi.org/10.1111/j.1745-6584.2012.00976.x>.

Hunt, R.J., L. Duncan, C. Haugh, J.T. White and J. Doherty. 2021. MODFLOW-NWT models and calibration files for the Mississippi Alluvial Plain, USA: U.S. Geological Survey data release. <https://doi.org/10.5066/P9AR7Y02>.

Leaf, A.T., M.N. Fienen and H.W. Reeves. 2021. SFRmaker and Linesink-maker: Rapid construction of streamflow routing networks from hydrography data. *Groundwater* <https://doi.org/10.1111/gwat.13095>.

McKenna, S. A., A. Akhriev, D.E. Ciaurri, and S. Zhuk. 2019. Efficient uncertainty quantification of reservoir properties for parameter estimation and production forecasting. *Mathematical Geosciences* 52: 1–19.

Miller, B.V., R.F. Adams, S.J. Stocks, J.L. Wilson, D.C. Smith and W.H. Kress. 2018. Waterborne resistivity surveys for streams in the Mississippi Alluvial Plain, 2017: U.S. Geological Survey data release. <https://doi.org/10.5066/F71J98ZQ>.

Miller, B.V., D.S. Wallace and W.H. Kress. 2016. Water-borne continuous resistivity profiling data from select streams of the Mississippi Alluvial Plain in northwestern Mississippi: U.S. Geological Survey data release. <https://doi.org/10.5066/F7FT8J68>.

Niswonger, R.G., S. Panday, and M. Ibaraki. 2011. MODFLOW-NWT, A Newton formulation for MODFLOW-2005: U.S. Geological Survey Techniques and Methods 6-A37, 44 p.

Westenbroek, S.M., J.A. Engott, V.A. Kelson and R.J. Hunt. 2018. SWB Version 2.0—A Soil-Water-Balance code for estimating net infiltration and other water-budget components: U.S. Geological Survey Techniques and Methods 6-A59, 118 p. <https://doi.org/10.3133/tm6A59>.

Westenbroek, S.M., M.G. Nielsen and D.E. Ladd. 2021a. OFR 2020-1008 MODEL ARCHIVE: Soil-Water-Balance model developed to simulate net infiltration and irrigation water use for the Mississippi Embayment Regional Aquifer System, 1915 to 2018. <https://doi.org/10.5066/P98PBR8O>.

Westenbroek, S.M., M.G. Nielsen and D.E. Ladd. 2021b. OFR 2020-1008 MODEL OUTPUT: Soil-Water-Balance net infiltration and irrigation water use output datasets for the Mississippi Embayment Regional Aquifer System, 1915 to 2018. <https://doi.org/10.5066/P9U484X5>.

Westenbroek, S.M., M.G. Nielsen and D.E. Ladd. 2021c. Initial estimates of net infiltration and irrigation water use from a Soil-Water-Balance model of the Mississippi Embayment Regional Aquifer System: U.S. Geological Survey Open File Report 2021-1008. 27 p. <https://doi.org/10.3133/ofr1008>.

Westenbroek, S.M., B.J. Dietsch and B.K. Breaker. 2021d. mapRandomForest— Monthly flow estimation in the Mississippi Alluvial Plain by means of Random Forest Modeling: U.S. Geological Survey software release, R package, Reston, Virginia, <https://doi.org/doi:10.5066/P92UE6EG>.

Westenbroek, S.M., B.J. Dietsch and B.K. Breaker. 2021e. Input and datasets and random forest model outputs for predicting stream flow and baseflow for the Mississippi Alluvial Plain, U.S. Geological Survey data release, Reston, Virginia, <https://doi.org/10.5066/P9QCK8HY>.

White, J.T., R.J. Hunt, M.N. Fienen, M.N., and J.E. Doherty. 2020. Approaches to Highly Parameterized Inversion: PEST++ Version 5, a Software Suite for Parameter Estimation, Uncertainty Analysis, Management Optimization and Sensitivity Analysis: U.S. Geological Survey Techniques and Methods, Book 7, Section C, Chapter 26. 52 p. <https://doi.org/10.3133/tm7C26>.

Wilson, J.L, 2020. Aquaculture and Irrigation Water-Use Model (AIWUM) Version 1 with Demonstration for the Mississippi Alluvial Plain: U.S. Geological Survey software release. <https://doi.org/10.5066/P9YDGJ7L>.
